# Supplementary material for: Engineered Mycobacterium tuberculosis triple-kill-switch strain provides controlled tuberculosis infection in animal models
Source: Nat Microbiol. 2025 Jan 10;10(2):482–94. doi: 10.1038/s41564-024-01913-5 (PMC11790485; doi:10.1038/s41564-024-01913-5)
Supplement: Supplementary file 1 — Reporting Summary [file 41564_2024_1913_MOESM1_ESM.pdf]

nature portfolio

Corresponding author(s): Eric J. Rubin

Last updated by author(s): Dec 4 2024

Reporting Summary

Nature Portfolio wishes to improve the reproducibility of the work that we publish. This form provides structure for consistency and transparency in reporting. For further information on Nature Portfolio policies, see our Editorial Policies and the Editorial Policy Checklist.

Statistics

For all statistical analyses, confirm that the following items are present in the figure legend, table legend, main text, or Methods section.

n/a	Confirmed	
☐	☒	The exact sample size (*n*) for each experimental group/condition, given as a discrete number and unit of measurement
☐	☒	A statement on whether measurements were taken from distinct samples or whether the same sample was measured repeatedly
☐	☒	The statistical test(s) used AND whether they are one- or two-sided<br>*Only common tests should be described solely by name; describe more complex techniques in the Methods section.*
☒	☐	A description of all covariates tested
☐	☒	A description of any assumptions or corrections, such as tests of normality and adjustment for multiple comparisons
☐	☒	A full description of the statistical parameters including central tendency (e.g. means) or other basic estimates (e.g. regression coefficient) AND variation (e.g. standard deviation) or associated estimates of uncertainty (e.g. confidence intervals)
☐	☒	For null hypothesis testing, the test statistic (e.g. *F*, *t*, *r*) with confidence intervals, effect sizes, degrees of freedom and *P* value noted<br>*Give P values as exact values whenever suitable.*
☒	☐	For Bayesian analysis, information on the choice of priors and Markov chain Monte Carlo settings
☒	☐	For hierarchical and complex designs, identification of the appropriate level for tests and full reporting of outcomes
☐	☒	Estimates of effect sizes (e.g. Cohen's *d*, Pearson's *r*), indicating how they were calculated

Our web collection on statistics for biologists contains articles on many of the points above.

Software and code

Policy information about availability of computer code

Data collection	No computer code was used in data collection.
Data analysis	Data were analyzed and visualized by GraphPad Prism 10. The whole genomic sequencing data was analyzed by Sickle (version 1.33), bwa (version 0.7.17), samtools (version 1.11-9) and VarScan (2.3.9). Flow cytometry was analyzed via FlowJo (version 10.8). Bacteria morphology imaging was analyzed via python package MOMIA (0.0.1).

For manuscripts utilizing custom algorithms or software that are central to the research but not yet described in published literature, software must be made available to editors and reviewers. We strongly encourage code deposition in a community repository (e.g. GitHub). See the Nature Portfolio guidelines for submitting code & software for further information.

Data

Policy information about availability of data

All manuscripts must include a data availability statement. This statement should provide the following information, where applicable:

- Accession codes, unique identifiers, or web links for publicly available datasets
- A description of any restrictions on data availability
- For clinical datasets or third party data, please ensure that the statement adheres to our policy

All relevant data generated in this study are present within the manuscript and Supplemental Information. Whole genome sequencing data for escape mutants of dual-lysin strain, ddTMP strain and TKS strain will be available on SRA, and the project and accession numbers will be listed prior to publication.

Research involving human participants, their data, or biological material

Policy information about studies with human participants or human data. See also policy information about sex, gender (identity/presentation), and sexual orientation and race, ethnicity and racism.

Reporting on sex and gender	N/A
Reporting on race, ethnicity, or other socially relevant groupings	N/A
Population characteristics	N/A
Recruitment	N/A
Ethics oversight	N/A

Note that full information on the approval of the study protocol must also be provided in the manuscript.

Field-specific reporting

Please select the one below that is the best fit for your research. If you are not sure, read the appropriate sections before making your selection.

☒ Life sciences ☐ Behavioural & social sciences ☐ Ecological, evolutionary & environmental sciences

For a reference copy of the document with all sections, see nature.com/documents/nr-reporting-summary-flat.pdf

Life sciences study design

All studies must disclose on these points even when the disclosure is negative.

Sample size	No statistical methods were used to pre-determine sample sizes but our sample sizes are similar to those reported in previous publications (PMIDs: 24315099, 34269789).
Data exclusions	No data were excluded from analysis.
Replication	All experiments contain biological replicates. All growth curve experiments, western blots and flow cytometry studies were repeated >2 times. All replicate experiments reproduced the original results.
Randomization	Mice and NHPs used in this study were randomly assigned into groups.
Blinding	Microscopy samples were de-identified prior to imaging. Blinding was not applicable due to experiment design,

Reporting for specific materials, systems and methods

We require information from authors about some types of materials, experimental systems and methods used in many studies. Here, indicate whether each material, system or method listed is relevant to your study. If you are not sure if a list item applies to your research, read the appropriate section before selecting a response.

Materials & experimental systems

n/a	Involved in the study	
☐	☒	Antibodies
☒	☐	Eukaryotic cell lines
☒	☐	Palaeontology and archaeology
☐	☒	Animals and other organisms
☒	☐	Clinical data
☒	☐	Dual use research of concern
☒	☐	Plants

Methods

n/a	Involved in the study	
☒	☐	ChIP-seq
☐	☒	Flow cytometry
☒	☐	MRI-based neuroimaging

Antibodies

Antibodies used	**anti-myc** (71D40, cell signaling); **anit-RpoB** (8RB13, cell signaling); **anti-CD16/32** (101302, Biolegend), **anti-CD69-BB700** (HI.2F3, BD Biosciences); **anti-CD44-FITC** (IM7, Biolegend), **anti-CD62L-BV650** (MEL14, BD Biosciences), **anti-CD11a-BV605** (2D7, 424 BD Biosciences), **anti-KLRG1-PE-Cy7** (2FI, Biolegend), **anti-PD-1-BV421** (29F.1AI2, Biolegend), **anti-CD8-425-BUV496** (53-6.7, BD

	Biosciences), **anti-CD3-BUV395** (17A2, BD Biosciences), **anti-CD4-APC-H7 426** (GKl.5, BD Biosciences), **anti-CD45-APC** (30-Fll, Biolegend), **IRDye® 680RD goat IgG (H + L) anti-mouse** (926-68070, LICORbio), **IRDye® 800CW goat IgG (H + L) anti-rabbit** (926-68071, LICORbio).
Validation	Antibodies were validated by the manufacturer. We also validated antibody via protein size for western blot and positive controls in flow cytometry.

Animals and other research organisms

Policy information about studies involving animals; ARRIVE guidelines recommended for reporting animal research, and Sex and Gender in Research

Laboratory animals	Mouse: C57BL/6J mice (The Jackson Laboratory, 000664); B6.Cg-Prkdcscid/SzJ mice (The Jackson Laboratory, 001913); B6.129S7-Ragltm1Mom/J mice (The Jackson Laboratory, 002216). All mice are 6- to 8-week old.<br>Nonhuman primates: 6-9 years old Mauritian cynomolgus macaques (Maccaca Fascicularis), obtained from Bioculture Mauritius.
Wild animals	No wild animals were used in this study.
Reporting on sex	All mice were female. All NHP were male.
Field-collected samples	No field collected samples were used in this study.
Ethics oversight	For mouse experiments, animal care and experimental procedures were conducted with the approval of the Institutional Animal Care and Use Committees (IACUC) of Harvard Medical School, Harvard T.H. Chan School of Public Health and Weill Cornell Medicine.<br><br>For NHP studies, all experimental manipulations, procedures, protocols, and care of the animals were approved by the University of Pittsburgh School of Medicine Institutional Animal Care and Use Committee (IACUC). The protocol assurance number for our IACUC is A3187-01. The specific protocol approval number is 18124087. The University of Pittsburgh's IACUC adheres to national guidelines established in the Animal Welfare Act (7 U.S.C. Sections 2131–2159) and the Guide for the Care and Use of Laboratory Animals (eighth edition), as mandated by the U.S. Public Health Service Policy.

Note that full information on the approval of the study protocol must also be provided in the manuscript.

Plants

Seed stocks	*Report on the source of all seed stocks or other plant material used. If applicable, state the seed stock centre and catalogue number. If plant specimens were collected from the field, describe the collection location, date and sampling procedures.*
Novel plant genotypes	*Describe the methods by which all novel plant genotypes were produced. This includes those generated by transgenic approaches, gene editing, chemical/radiation-based mutagenesis and hybridization. For transgenic lines, describe the transformation method, the number of independent lines analyzed and the generation upon which experiments were performed. For gene-edited lines, describe the editor used, the endogenous sequence targeted for editing, the targeting guide RNA sequence (if applicable) and how the editor was applied.*
Authentication	*Describe any authentication procedures for each seed stock used or novel genotype generated. Describe any experiments used to assess the effect of a mutation and, where applicable, how potential secondary effects (e.g. second site T-DNA insertions, mosiacism, off-target gene editing) were examined.*

Flow Cytometry

Plots

Confirm that:

☒ The axis labels state the marker and fluorochrome used (e.g. CD4-FITC).

☒ The axis scales are clearly visible. Include numbers along axes only for bottom left plot of group (a 'group' is an analysis of identical markers).

☒ All plots are contour plots with outliers or pseudocolor plots.

☒ A numerical value for number of cells or percentage (with statistics) is provided.

Methodology

Sample preparation	Mtb flow cytometry: Mtb cells were fixed in 2% paraformaldehyde overnight and removed from the BSL-3 facility. 331 Fixed bacilli were quenched with 200 mM Tris-HCl (pH 7.5) for 5 minutes at room temperature 332 and resuspended in PBST buffer (1x PBS with 0.1% Triton X-100). To suppress signals from 333 noise or cell debris, two event triggers (thresholds) on forward scatter peak height (FSC-H >1.5) 334 and side scatter area (SSC-A > 1.0) were used upon recording.

For lung cell flow cytometry: Lung cell suspensions were washed with PBS twice, stained for viability with the live/dead dye Zombie UV (Biolegend, 423107) for 10 min at 4°C, washed with Cell Staining Buffer (BioLegend 420201) and incubated with Fc block (purified anti-mouse CD16/CD32 antibody, Biolegend 101302) at 1:200 in Cell Staining Buffer for 10 min at 4°C. After one wash in Cell Staining Buffer, cells were incubated with fluorochrome-conjugated monoclonal antibodies (mAbs) diluted at 1:200 into a 1:3 solution of Brilliant Staining Buffer (BD 563794): Cell Staining Buffer, for 45 min at 4°C. Cells were then washed twice in Cell Staining Buffer and fixed with Fixation Buffer (Biolegend 420801) for 30 min at 4°C. In all incubation steps cells were protected from light. Fluorescence minus one (FMO) controls were stained alongside samples.

Instrument	Mtb flow cytometry: MACSQuant Analyzer 10 flow cytometer (Miltenyi Biotec)<br>Lung flow cytometry: FACSymphony A5 Cell Analyzer (BD Biossciences)
Software	FlowJo v10.8 (BD Life Sciences) was used for flow cytometry analysis.
Cell population abundance	The final cell/bacteria population was 0.5 to 20% of total events.
Gating strategy	Mtb flow cytometry: To suppress signals from noise or cell debris, two event triggers (thresholds) on forward scatter peak height (FSC-H >1.5) and side scatter area (SSC-A > 1.0) were used upon recording. To remove cellular aggregation, stringent gate settings were manually defined via FlowJo v. 10.8 to exclude events with strongly correlated forward scatter area (FSC-A) and SSC-A measures (large and compact particles), as well as events with disproportional FSC-A and FSC-H measures (morphological outliers). After event filtration, the log10-transformed red fluorescence intensity peak height (denoted TdTomato-A) was used to represent the abundance of intracellular red fluorescence protein.<br><br>Lung cytometry: The gating strategy is shown in supplement figure 1.

☒ Tick this box to confirm that a figure exemplifying the gating strategy is provided in the Supplementary Information.
