# Supplementary figures and images for: Engineered Mycobacterium tuberculosis triple-kill-switch strain provides controlled tuberculosis infection in animal models
Source: Nat Microbiol. 2025 Jan 10;10(2):482–94. doi: 10.1038/s41564-024-01913-5 (PMC11790485; doi:10.1038/s41564-024-01913-5)

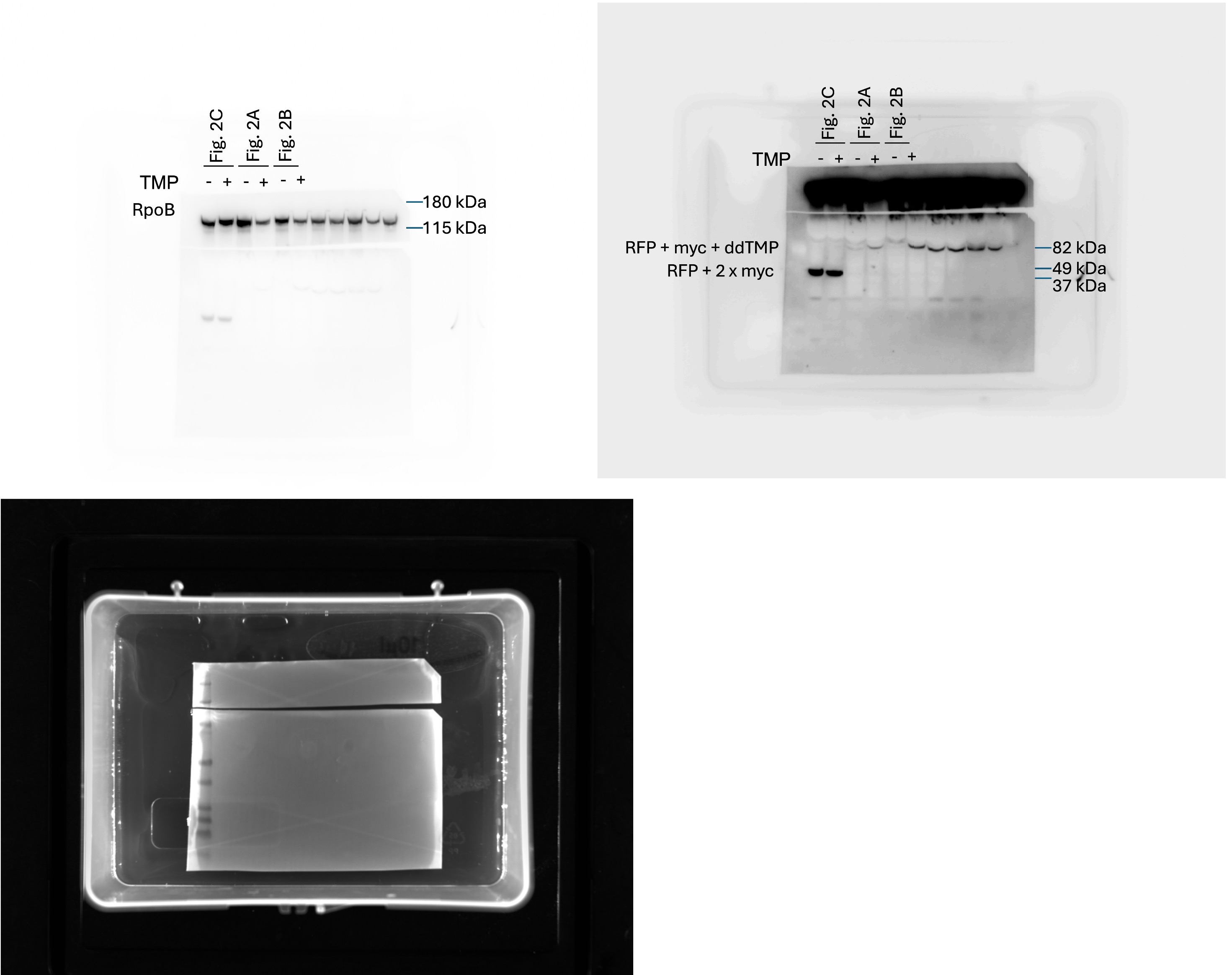

Supplement: Supplementary file 4 — Unprocessed western blots of Fig. 2a–c. [file 41564_2024_1913_MOESM4_ESM.jpg]

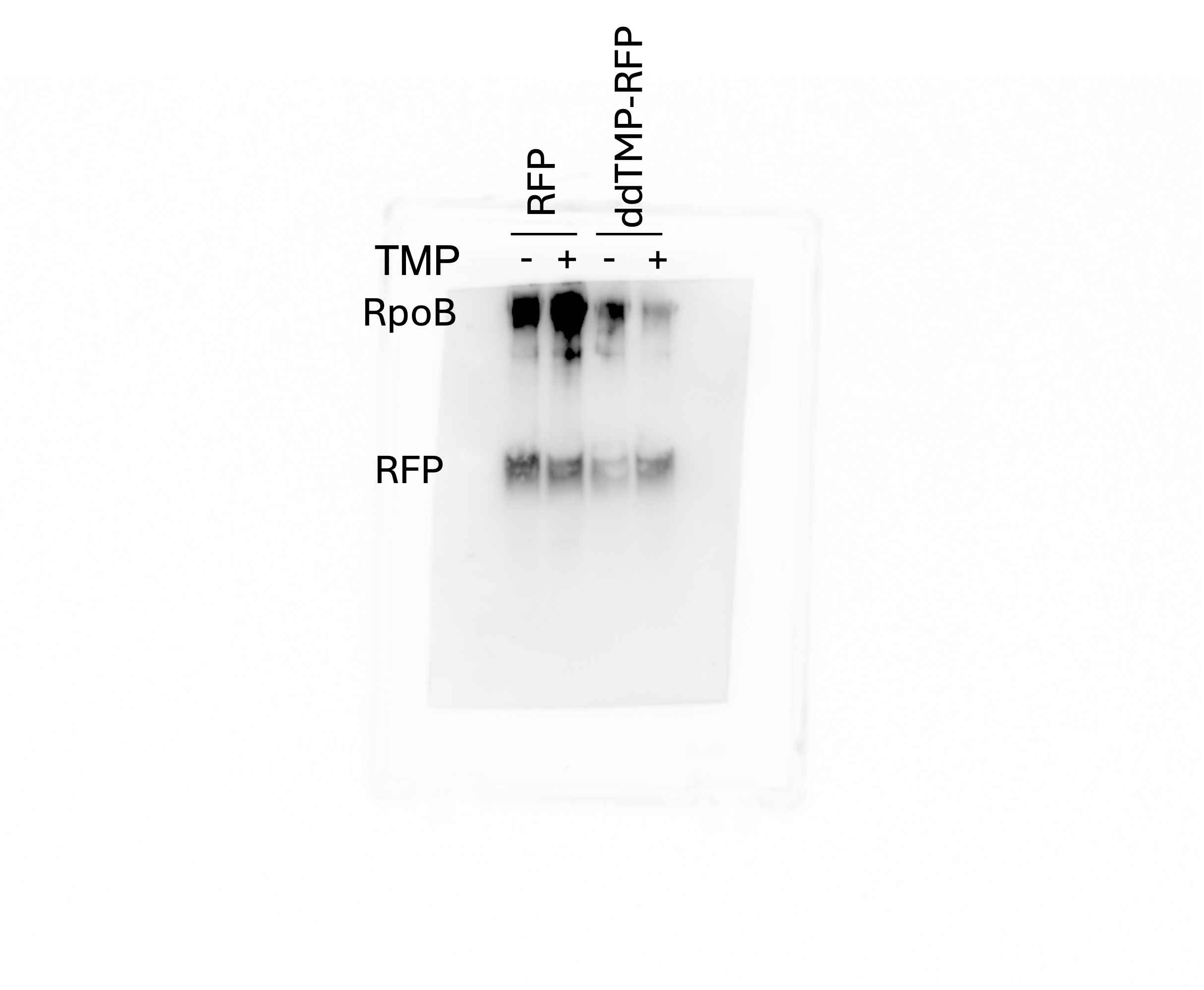

Supplement: Supplementary file 5 — Unprocessed western blots of Fig. 2d. [file 41564_2024_1913_MOESM5_ESM.jpg]
